# Editorial: Global excellence in fungal pathogenesis: Asia & Australasia

**DOI:** 10.3389/fcimb.2025.1573927

**Published:** 2025-03-04

**Authors:** Shweta Panchal, Zhangyong Si

**Affiliations:** ^1^ School of Bio Sciences and Technology, Vellore Institute of Technology, Vellore, India; ^2^ Ningbo Institute of Materials Technology and Engineering, Chinese Academy of Sciences, Ningbo, China

**Keywords:** fungal pathogens, *Candida*, *Aspergillus*, virulence, drug resistance

Fungi are an integral part of our ecosystem, playing key roles in decomposition and nutrient cycling. However, a subset of fungal species, including *Candida auris*, *Aspergillus fumigatus*, and *Cryptococcus neoformans*, has emerged as significant pathogens, specially in immunocompromised individuals. Among global health challenges, fungal diseases have often been ignored. While bacterial and viral infections frequently capture the headlines, fungi, despite their ubiquity, are an overlooked yet significant threat to human health. The World Health Organization (WHO) has recently flagged fungal infections as a rising global concern and has released a list of critical priority fungal strains in 2022 that represent the greatest threat to global infection (World Health Organization, 2022).

The rise of invasive fungal infections is particularly alarming. For instance, *Candida auris* has emerged as one of the top priority fungal pathogen owing to its ability to resist multiple antifungal drugs, survive on surfaces for extended periods, and cause outbreaks in healthcare settings. Similarly, infections caused by *Aspergillus fumigatus* are becoming harder to treat as many strains have displayed resistance to azole antifungals, which are widely used in both medicine and agriculture (Hui et al., 2024). The increasing prevalence of fungal diseases is exacerbated by factors such as climate change, global trade, and the emergence of drug-resistant strains (Casadevall et al., 2019; Nnadi and Carter, 2021). Rising global temperatures are expanding the habitats of pathogenic fungi, enabling them to thrive in regions previously deemed inhospitable. For example, *Coccidioides* spp., responsible for Valley fever, is increasingly reported in areas beyond its traditional endemic zones in the southwestern United States. Furthermore, the adaptability of fungi to warmer temperatures poses a unique challenge. Some species are evolving to survive at the human body’s core temperature, a trait previously thought to limit their pathogenic potential. This evolutionary shift underscores the urgency of monitoring fungal behavior in a warming world.

This Research Topic aimed to highlight some recent views and innovations in diverse aspects of fungal pathogenesis. Jafarlou‘s review provides a comprehensive overview of fungal pathogens with the potential to cause pandemics, including *C. auris*, *A. fumigatus*, *C. neoformans*, *Histoplasma capsulatum*, *Pneumocystis jirovecii*, and Mucoromycetes. It discusses their virulence mechanisms and the factors that may contribute to their pandemic potential. *Candida auris* has recently gained lot of attention due to sudden appearance of hospital-infection causing strains in different parts of the world, with many being multidrug resistant isolates (Lockhart et al., 2017; Casadevall et al., 2019). While it shares many features with other species *Candida* group, scientists are now studying the specific virulence factors that may give these strains an extra edge over other species. In this Research Topic, Kim et al. demonsrate one such virulence factor to be a hydrolytic enzyme belonging to secreted aspartyl proteinase family, SAP3, which is important for virulence in *C. auris* and hence could be a relevant drug target, especially in drug resistant strains of *C. auris*.

Timely and accurate diagnosis of fungal infections is key to timely treatment and reduction in mortality. While culture-based techniques are most commonly used for fungal infections, newer molecular techniques, specifically based on PCR are being introduced. Wang et al. have developed a novel diagnostic tool, ERT-LAMP-CA, for the rapid and ultrasensitive detection of *Candida albicans* infections. This method integrates loop-mediated isothermal amplification (LAMP), restriction endonuclease cleavage, and real-time fluorescence detection, allowing for diagnosis within approximately one hour.

Due to the evolution of drug-resistant strains, scientists are delving into improving treatment options for fungal infections. In this Research Topic, Gao et al. provide the evidence for combination therapy involving azoles and proton-pump inhibitors (PPIs) to treat azole-resistant infections of *Candida* and *Aspergillus* species. They show that PPIs may reverse azole resistance, offering new strategies for treating invasive fungal infections. Feng et al. provide a comprehensive overview of immunotherapy targets for treating candidiasis and highlight the potential of vaccines and monoclonal antibodies to enhance immune responses against *C. albicans*, offering a promising avenue for treatment.

In conclusion, this Research Topic has a diverse collection of articles shedding light on the pathogenesis, diagnosis and treatment options for critical fungal pathogens.

## References

[B1] Casadevall A. Kontoyiannis D. P. Robert V. (2019). On the emergence of *Candida auris*: climate change, azoles, swamps, and birds. mBio. 10, 41–47. doi: 10.1128/mBio.01397-19 PMC6650554 31337723

[B2] Hui S. T. Gifford H. Rhodes J. (2024). Emerging antifungal resistance in fungal pathogens. Curr. Clin. Micro. Rpt. 11, 43–50. doi: 10.1007/s40588-024-00219-8 PMC11076205 38725545

[B3] Lockhart S. R. Etienne K. A. Vallabhaneni S. Farooqi J. Chowdhary A. Govender N. P.. (2017). Simultaneous emergence of multidrug resistant *Candida auris* on three continents confirmed by whole genome sequencing and epidemiological analyses. Clin. Infect. Dis. 64, 134–140. doi: 10.1093/cid/ciy333 27988485 PMC5215215

[B4] Nnadi N. E. Carter D. A. (2021). Climate change and the emergence of fungal pathogens. PloS Pathog. 17, e1009503. doi: 10.1371/journal.ppat.1009503 33914854 PMC8084208

[B5] World Health Organization (2022). WHO fungal priority pathogens list to guide research, development and public health action (Geneva: World Health Organization). Available at: https://www.who.int/publications/i/item/9789240060241 (Accessed October 25, 2022).

